# Laparoscopic partial cystectomy through extraperitoneal space for primary bladder schwannoma

**DOI:** 10.1186/s40001-022-00796-8

**Published:** 2022-09-06

**Authors:** Weihui Liu, Qingliu He, Xiaoping Su

**Affiliations:** Department of Urology, The Second Affiliated Hospital of Fujian Medical University, Quanzhou, 362000 Fujian China

**Keywords:** Bladder schwannoma, Laparoscopic partial cystectomy, Extraperitoneal space

## Abstract

**Background:**

Schwannomas can occur in the body where nerve sheaths are present. Genitourinary schwannomas are very rare, especially primary bladder schwannomas. They account for only 0.1% of bladder tumours. The literature reports that simple surgical resection has a good effect and prognosis.

**Case presentation:**

A 39-year-old man had no significant improvement in symptoms due to frequent urination and urgency for 1 month following the treatment of prostatitis for 2 weeks. Ultrasound and computed tomography (CT) showed a mass in the left side wall of the bladder (size approximately 2.0 × 1.9 cm) that had clear boundaries and protruded outward from the bladder. After the extraperitoneal space was dilated with a balloon, a minimally invasive laparoscopic partial cystectomy was performed in this space to remove the tumour. The pathological diagnosis was bladder schwannoma. Immunohistochemical staining showed that it was strongly S100 protein positive. There was no recurrence after 2 years follow-up by cystoscopy and CT.

**Conclusions:**

Bladder schwannomas are clinically rare benign bladder lesions and no specific clinical manifestations. Laparoscopic partial cystectomy through the extraperitoneal space is a safe and feasible treatment option.

## Background

Worldwide, the incidence of bladder cancer ranks 11th among malignant tumours. In Europe and the United States, the incidence of bladder cancer ranks 4th among male malignancies [1]. The most common form of bladder cancer is urothelial carcinoma (> 90%), followed by squamous cell carcinoma (3–7%), adenocarcinoma (< 2%) and other rare pathological types [2]. Bladder schwannomas are mesenchymal tumours, accounting for approximately 0.1% of all bladder tumours. They are currently considered to be benign tumours. Pathological diagnosis is the standard for the diagnosis of schwannomas [3]. Surgical resection, including partial cystectomy and transurethral resection of bladder tumour (TURBT), is effective for the treatment of schwannomas. We report a case of minimally invasive laparoscopic partial cystectomy through the extraperitoneal space and review previous literature.

## Case presentation

**Fig. 1:**
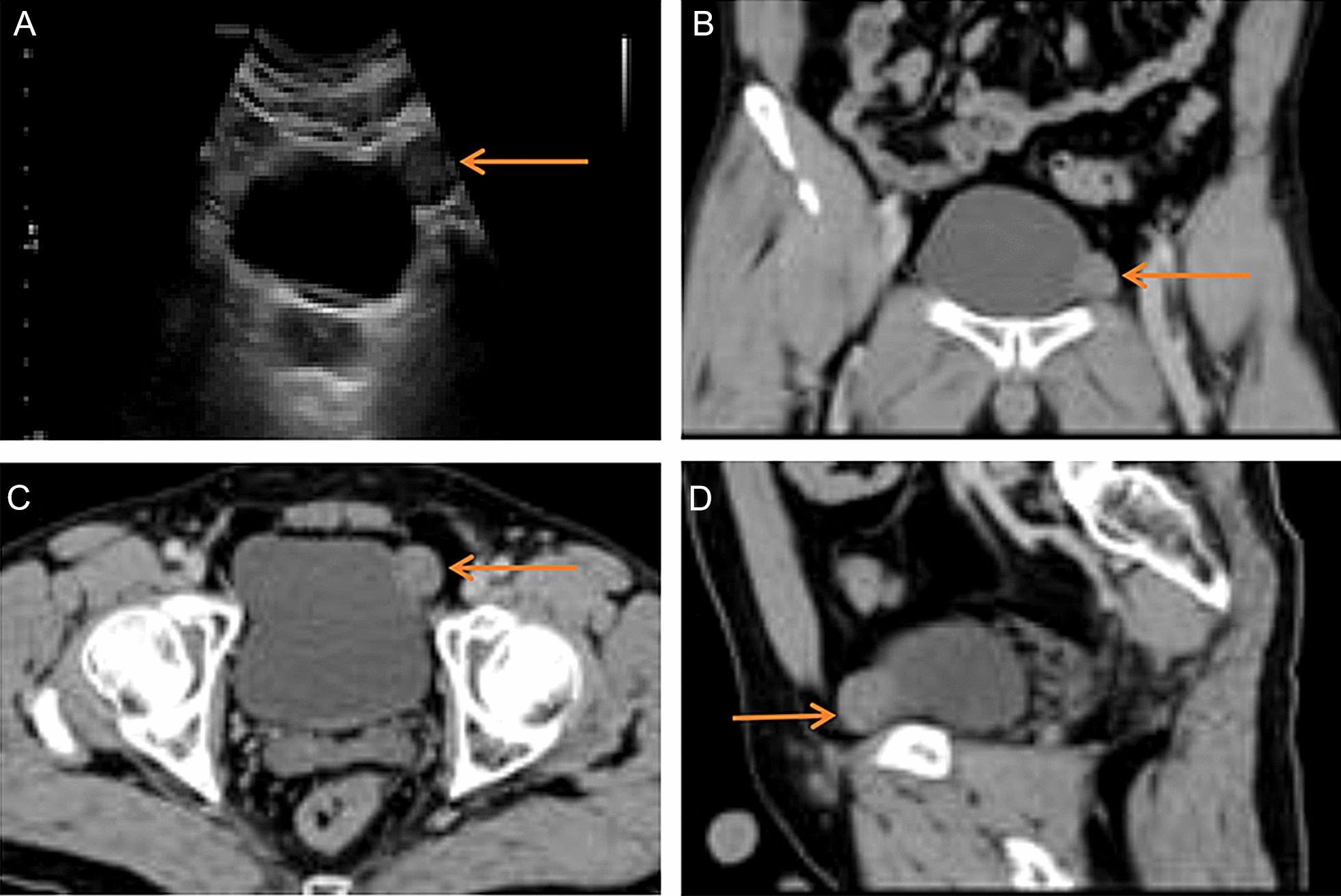
Imaging performance of bladder schwannoma **A** A solid lesion in the left upper part of the pelvic cavity by ultrasound. **B** A mass located in the left front wall of the bladder, coronal position CT scan value 53.7 HU. **C** CT cross-section. **D** CT sagittal position

The patient, a 39-year-old man with frequent urination, urgency and pain in the bladder area while holding back the urine for 1 month was in good health before. There was no abnormality in the urine routine. The examination of the prostatic fluid showed elevated white blood cells and decreased lecithin bodies. Prostatitis was initially diagnosed. After two weeks of treatment with cephalosporin and M receptor blocker, the symptoms of frequent urination and urgency were partially improved. Review the prostatic fluid indicated that the white blood cells were normal, and the lecithin bodies increased by 20% compared to 2 weeks ago, but the pain in the bladder area was not significantly relieved when the urine was held. Further examination of the urinary system color Doppler ultrasound suggest that the bladder had occupying space. An approximately 2.0 cm solid lesion in the left upper part of the pelvic cavity was seen by ultrasound (Fig. 1A). The admission-related examination, including blood, coagulation, liver function and kidney function tests as well as electrocardiogram and chest X-ray, showed no obvious abnormalities. CT suggested that a 2.0 × 1.9 cm mass was located in the left front wall of the bladder and was protruding outward with clear boundaries (Fig. 1B–D). There was no obvious abnormality of the bladder mucosa on cystoscopy. Nuclear heterogeneous cells were not detected in the urinary exfoliative cytology.

**Fig. 2:**
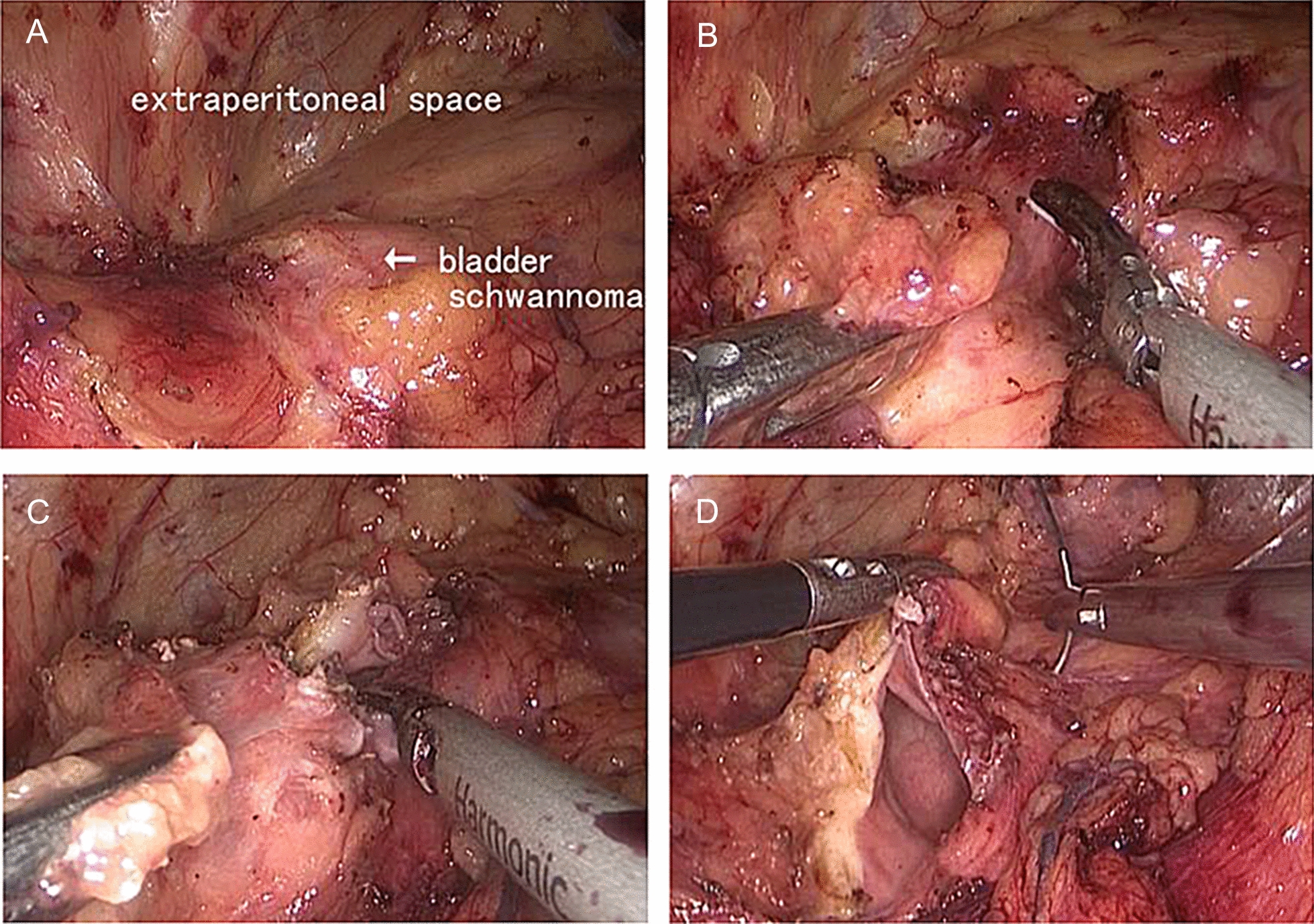
Laparoscopic partial cystectomy through extraperitoneal space. **A** Exposure of extraperitoneal space. **B** Isolation of tumour. **C** Resection of tumour. **D** Suture bladder suture

**Fig. 3:**
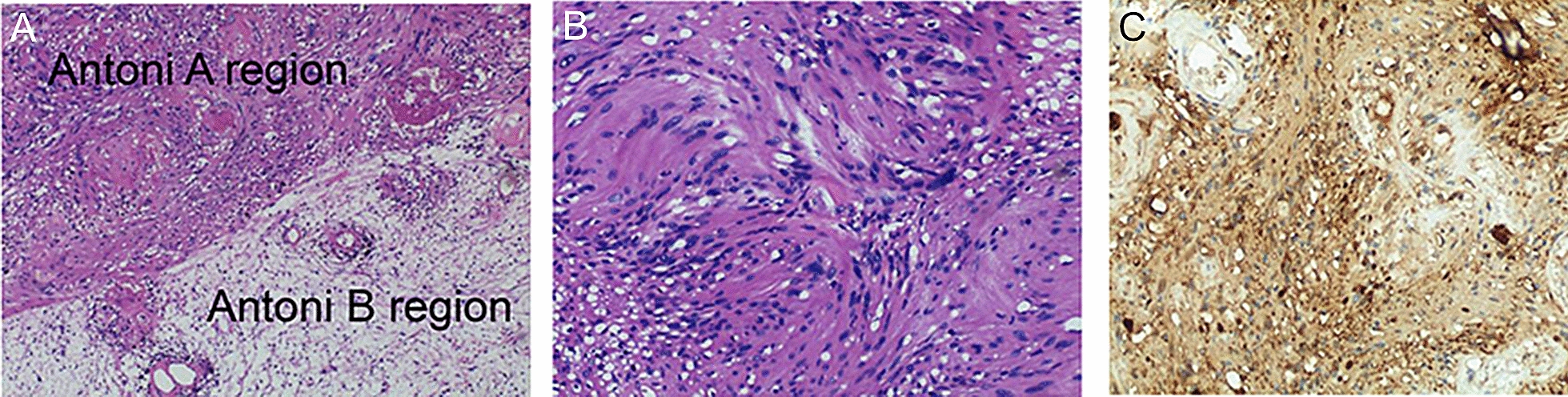
Pathology of bladder schwannoma. **A** Antoni A and B region in bladder schwannoma tissue (× 40). **B** Bladder schwannoma, spindle cells in a large number of mucus—like stroma, HE staining (× 100). **C** S100 is mainly expressed on the nucleus and cytoplasm, the membrane is not stained (× 100)

With the results of the imaging examination, a benign tumour was considered. Based on the tumour being convex to the outside of the bladder, a laparoscopic partial cystectomy through the extraperitoneal space was performed (Fig. 2). After general anaesthesia, the patient was placed in the supine position with the buttocks raised by 10 cm. We selected a 3 cm midline incision under the umbilicus as the observation hole, cutting the skin, subcutaneous tissue and the anterior and posterior sheaths of the rectus abdominis layer by layer. Placing the balloon under the posterior rectus abdominis, the extraperitoneal space was expanded (Fig. 2A). We separated the adipose tissue on the anterior wall of the bladder. A mass, 2.0 × 2.0 cm in size with a smooth surface, was seen on the left anterior wall of the bladder and protruded outside of the bladder. Using an ultrasonic knife, the tumour was completely removed from the bladder with 0.5 cm normal bladder tissue margins (Fig. 2B, C). The bladder wall was sutured by using 1–0 absorbable barbed suture through continuous full-thickness (Fig. 2D). A total of 200 ml of normal saline was injected into the bladder through the catheter, and there was no fluid extravasation in the incision of the bladder wall, which confirmed that the suture was reliable. The operation lasted 65 min, and the intraoperative blood loss was approximately 20 ml. Pathological examination described that the bladder mass had a size of 2 × 2 × 1.6 cm, with a surface covering of bladder mucosa and an envelope with clear boundaries, while the cut surface was greyish white. A schwannoma with oedema and bleeding was considered by pathological diagnosis. We used Envision staining to detect the expression of biomarkers. Immunohistochemical staining showed: S100(+), Desmin(−), SMA(−), CD34(−), Ki67 (about 8%+) (Fig. 3). The patient used a semi-liquid diet and walked after getting out of bed on the 1st day after surgery, without obvious gastrointestinal symptoms such as abdominal distension. The pelvic drainage tube was removed on the 4th day after surgery, and the catheter was removed on the 7th day after surgery. There was no recurrence after 2 years follow-up by cystoscopy and CT.

## Discussion and conclusions

Schwannomas are derived from Schwann cells, which are often associated with von Recklinghausen disease, but the relationship between bladder schwannomas and this disease is not clear [4]. Bladder schwannomas, which may originate from the autonomic plexus or the ganglia of the bladder and urethra, are a rare type of bladder tumour in clinical practice, and they also occur in the adrenal glands, seminal vesicles and scrotum [5, 6]. There were 12 English studies retrieved from 1993 to present (Table 1). There were 13 cases (6 males and 7 females), with an age range of 19 to 88 years old and A maximum tumour diameter of 0.7–20 cm. The clinical manifestations of bladder schwannomas are mostly painless gross haematuria with lower urinary tract symptoms (LUTS) [3, 7–11], abdominal pain [4, 8] and urinary tract infection [7, 12]. Prostatitis is a common disease in urology. The symptoms are chronic pelvic pain syndrome, such as frequent urination, urgency, perineal pain, etc. It can be diagnosed by combining urine routine and prostatic fluid examination. Usually use antibiotics, alpha or M receptor block therapy. The male patient showed no significant improvement after treatment, and was diagnosed with bladder space-occupying lesions by color Doppler ultrasound and CT. Ultrasonography can be used as the preferred method for the diagnosis of space-occupying lesions of the bladder, but it cannot accurately describe the degree of infiltration of the mass and surrounding tissues and organs. The tumours represented by CT are solitary, encapsulated and non-calcified in the bladder wall, while on enhanced scanning, tumours can be expressed in the parenchyma obviously, with a small amount of swelling and strengthening of tumour blood vessel shadows with clear boundaries [13]. For our case, CT performance was similar to that reported in the literature. Although magnetic resonance (MR) is considered to be more sensitive than CT for the diagnosis of schwannomas [14], the CT diagnosis of this case considered leiomyoma or schwannoma, which was effectively differentiated from bladder cancer. Pathological examination is the standard for the diagnosis of bladder schwannomas. Schwannomas has two kinds of tissue structure: Antoni A region, which is also called bundle-shaped area. The Antoni B region, the cell loosening zone, also known as the reticular zone. Immunohistochemical staining tests with S100 positive expression are localized in the nucleus and cytoplasm and are considered to be a specific biomarker. Antoni A and B region observed with S100 positive expression are important features in the diagnosis of schwannomas [7, 15, 16]. Two cases did not detect S100, because HE staining of tumour tissue suggested a typical schwannoma [9, 11]; the other 11 cases were S100 positive. Bladder schwannomas are benign tumours with slow growth. Surgical resection is the main method of treatment. The methods include partial cystectomy [4, 7–9, 12] and TURBT [3, 10, 11, 17, 18]. All of the cases reported for partial cystectomy are open, except for a case report of laparoscopic partial cystectomy [13]. The tumour should be completely resected. Follow-up observation after pathological diagnosis was considered as another treatment method instead of resection [19]. There was no recurrence after 1 to 48 months of follow-up after surgery in previous literature. The bladder is an inter-retroperitoneal viscera. We used laparoscopic partial cystectomy with an extraperitoneal approach to completely remove the tumour. Because the operation was performed through the extraperitoneal space and without incision of the peritoneum, there were no intestinal-related complications after the operation. Theoretically, benign tumours have the potential to change to malignancies. We recommend active surgery as the primary treatment for bladder schwannomas. When the tumour is located in the anterior or lateral wall of the bladder and protrudes out of the bladder, laparoscopic partial cystectomy through the extraperitoneal space is safe and feasible. To our knowledge, this is the first case of laparoscopic partial cystectomy for the treatment of bladder schwannoma in the extraperitoneal space. Compared with open surgery and laparoscopic partial cystectomy, laparoscopic partial cystectomy through the extraperitoneal space has the following advantages: first, the operation space is large enough; second, there is no abdominal cavity interference during the operation; and third, there are no intestinal-related complications after the operation, making the recovery fast and hospital stay short. After the bladder tumor was removed, the patient's symptoms of frequent urination, urgency and lower abdominal pain disappeared.

**Table 1:** Previously reported cases of bladder schwannoma (sorted by year of publication)

No.	Authors/year	Age/sex	Presentation	Location of bladder	Maximum diameter (cm)	Treatment	Follow-up (months)	Expression of S100
[1]	Ng/1993 [19]	88/F	Incontinence	Left lateral wall	20.0	Biopsy	N/A	+
[2]	Brown/1997 [12]	19/F	Urinary tract infection	Medial to the left ureteric orifice	1.0	Partial cystectomy	18	+
[3]	Cummings/1998 [4]	58/F	Abdominal pain	Left lateral wall	4.5	Partial cystectomy	36	+
[4]	Geol/2005 [13]	35/M	None	Left lateral wall	3.5	Laparoscopic Partial Cystectomy	12	+
[5]	Wang/2008 [7]	69/M	Hematuria, recurrent infection	N/A	N/A	Partial cystectomy	48	+
[6]	Wang/2008 [7]	56/F	N/A	N/A	N/A	Partial cystectomy	48	+
[7]	Gafson/2008 [8]	52/F	LUTS, abdominal pain	Left anterior wall	7.0	Partial cystectomy	1	+
[8]	Mosier/2012 [9]	31/M	Hematuria	Left lateral wall	1.7	Partial cystectomy	8	Not tested
[9]	Mazdar/2014 [3]	50/F	Hematuria, LUTS	Postero-lateral right wall	5.8	TURBT	5	+
[10]	Srinivasa/2016 [10]	45/M	Hematuria	Dome of the bladder	1.6	TURBT	9	+
[11]	Jallad/2018 [17]	25/F	None	Left lateral wall	N/A	TURBT	6	+
[12]	Bakurov/2018 [11]	53/M	Hematuria, LUTS	Triangle area	3.5	TURBT	12	Not tested
[13]	Nasrollahi/2020 [18]	35/M	Frequent urination	Dome wall	0.7	TURBT	N/A	+
[14]	This case /2019	39/M	Frequent urination, urgency, pain	Left anterior wall	2.0	Laparoscopic partial cystectomy through extraperitoneal space	12	+

*N/A* not applicable *TURBT* transurethral resection of bladder tumor *LUTS* lower urinary tract symptoms

This article describes an extremely rare case of bladder schwannoma, which is a clinically rare benign bladder lesion. Its clinical manifestations are non-specific. Bladder schwannoma can not be diagnosed initially through clinical symptoms, but requires cystoscopy, color Doppler ultrasound and CT. Determining diagnosis base on histopathology, and S100 positive expression is a specific marker. Active surgical intervention is recommended, and its effect is satisfactory. Laparoscopic partial cystectomy through the extraperitoneal space is safe and feasible when the tumour is in the anterior wall and side wall of the bladder and protruding out of the bladder. Its long-term prognosis needs further follow-up study.

## Data Availability

Not applicable.

## Abbreviations

CT: Computed tomography
TURBT: Transurethral resection of bladder tumour
LUTS: Lower urinary tract symptoms
MR: Magnetic resonance
